# Cervical mature teratoma in pediatric

**DOI:** 10.1016/j.radcr.2022.08.106

**Published:** 2022-09-30

**Authors:** Serlly Wattimury, Lenny Violetta

**Affiliations:** Department of Radiology, Faculty of Medicine Universitas Airlangga - Dr. Soetomo Academic General Hospital, Jalan Mayjen. Prof. Dr. Moestopo 47, Surabaya 60131, Indonesia

**Keywords:** Mature teratoma, Cervical mass, Neck mass

## Abstract

Cervical teratomas are one of the rare tumors. Relating to the size of the tumor, they present as a huge neck mass with solid and cystic components. Furthermore, they are able to induce a hyperextension of the neck, neonatal respiratory distress, and possible malignancy. The computed tomography scan examination of this case revealed that there was a mass. It was a component of a teratoma and pathological anatomy which supported the finding. Thus, this study provided a case of a fully excised and cured cervical mature teratoma occurred in an infant. Surgical management must be undergone as thorough as feasible in order to prevent recurrences and the development of the cancer.

## Introduction

The term “teratoma” is coined from a Greek word which means a “monster.” Commonly, it is originated from the tissues of the ectoderm, mesoderm, and endoderm. These three germ cell layers serve as the basis [1]. Moreover, the sacrococcygeal region is the most frequent site for teratomas to be occurred, while the cervical region is a less frequent place [2]. On the other hand, histologically, cervical teratomas are considered as a benign. Relating to their location and size, they can trigger the airway obstruction [3]. The current case report places the particular emphasis on the rarity of cervical teratomas. Moreover, this study pointed out the challenges in using the fine-needle aspiration cytology of these tumors. These mechanisms served as the gold standard for a conclusive diagnosis of this entity.

## Case report

**Fig. 1:**
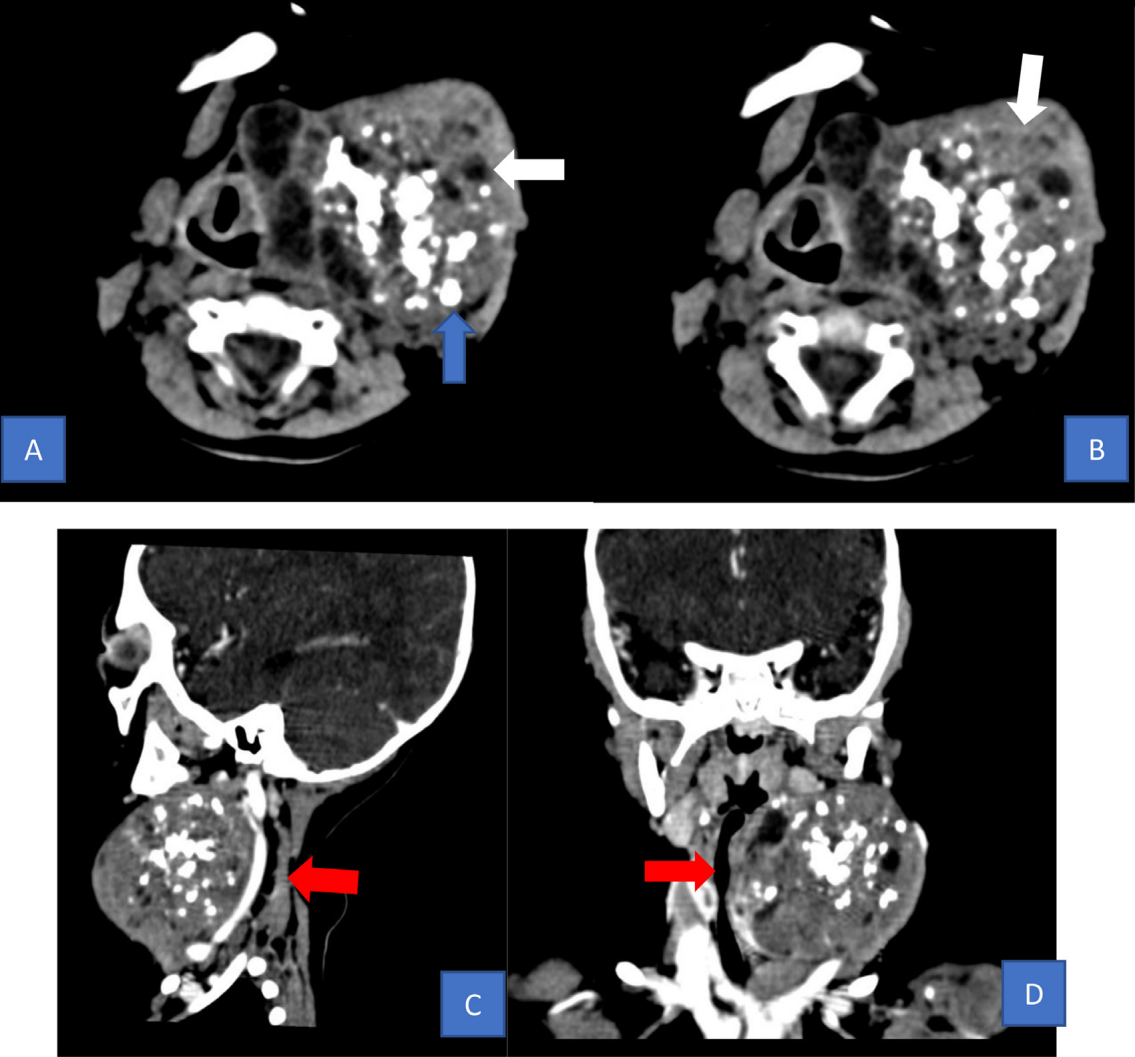
(A) CT scan of the left colli showed that there was a heterogeneous solid and cystic mass (white arrow) with lobulated borders that had calcifications (blue arrow). (B) An axial intravenous contrast-enhanced image showed no obvious enhancement. (C) The airway was neither compressed nor distorted (red arrow). (D) Coronal views in computed tomography.

A 7-month-old male infant presented to the surgical outpatient department with the prior complaints of the left-sided neck mass. This condition was presented since he was born. The swelling measured approximately in 5.6 × 7 × 7.6 cm. The infant was properly fed as in his normal sensory and motor milestones were well-indicated. There were no symptoms of respiratory distress. Moreover, the infant underwent a computed tomographic scan which resulted in; a complex heterogeneous solid cystic and calcification enhancing mass lesion in the left cervical region with extensions into the left parotid space, left buccal space, left submandibular space, left carotid space, left parapharyngeal space and left retropharyngeal space (Fig. 1). Furthermore, the possibility of cervical teratoma was diagnosed as preliminary and fine needle aspiration cytology was advised to be performed.

### Histopathological findings

**Fig. 2:**
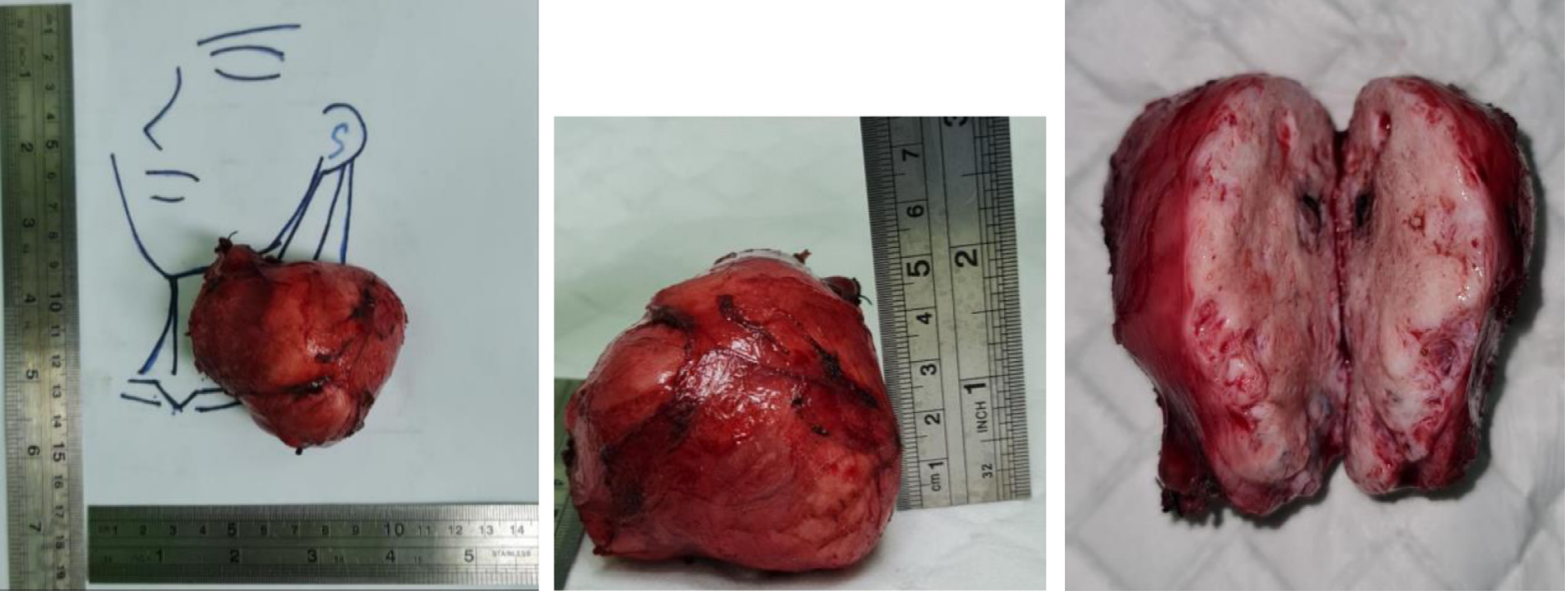
(A) Pictures during the surgical procedure showed that there was a typical appearance of the lobular soft tissue mass and (B) tumor totally removed.

**Fig. 3:**
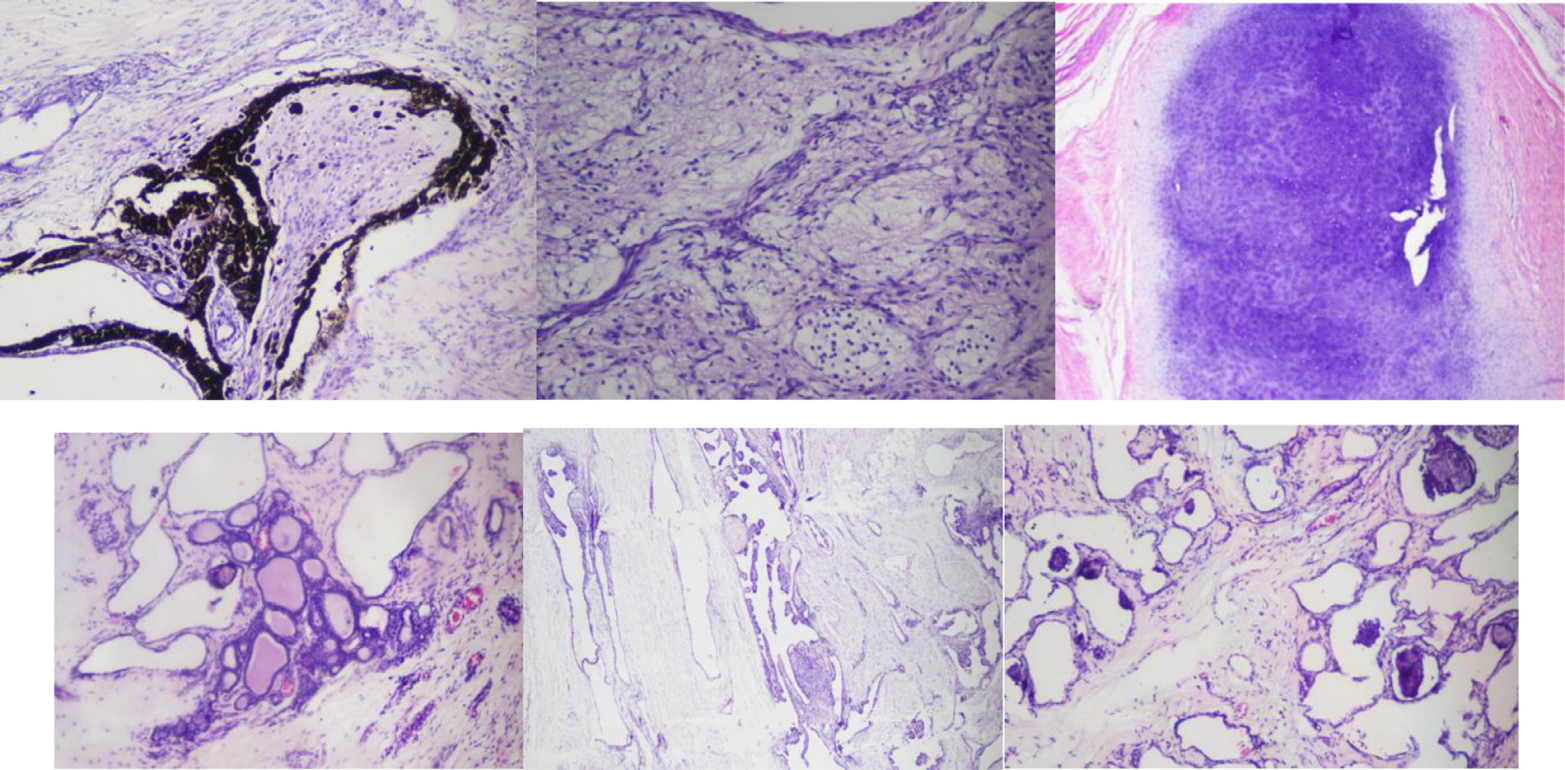
Microscopic examination revealed that there was a sebaceous glans, cartilage adipose, thyroid tissue, calcification area, choroid tissue, melanocyte pigment and trabeculation of bone.

Apparently, the tumor was found in a gray-white to brown with globular measuring as 6 × 5 × 4.5 cm. The cut section portrayed a variegated appearance with focal areas of cystic degeneration as in Figure 2. In addition, microscopic examination revealed a sebaceous glans, cartilage adipose, thyroid tissue, calcification area, choroid tissue, melanocyte pigment and trabeculation of bone. There was no euroephitelial component found (Fig. 3).

## Discussion

The most prevalent extragonadal germ cell tumors found in children are teratomas. There is no gender prevalence in these tumors. The typical one has an incidence of 4000 live births [1]. In addition, they have a risk of developing in other congenital abnormalities as many as 18% [1,2]. Only 5% of all teratomas, which are mostly occurred in the cervical, nasopharynx, face, and orbit, are found in the head and neck region. Based on the origin, cervical teratomas can be categorized as thyroid or extrathyroid [4]. The total incidence of cervical teratomas has been found to range from 2.3% to 9.3% [4].

Children's cervical teratomas are typically benign; however, they are occasionally aggressive. They may exhibit respiratory distress which the case of rapid excision is indicated. Relating to the radiological perspective, multiple calcified foci on computed tomography are very suggestive kind of teratomas [5]. CT examination is useful for determining the lesion's extent and intracranial extension [6]. Moreover, teratomas have been misinterpreted for meningoencephalocele in CT scanning. Intraoperative aspiration is properly advised before the excision has been performed. The diagnosis of teratoma is suggested through the mechanism of the calcifications on the plain film or other radiographic study. In terms of the ultrasound, cervical teratomas appear as a massive, multiloculated, multiseptated mass lesions with both solid and cystic components as well as scattered areas of calcification [7], [8], [9].

There are some differential diagnoses for teratomas, e.g., lymphangioma, venous malformations with phleboliths, dermoid, neurenteric cysts, Thornwald's cyst, and basal meningocele. All cervical teratomas should have preoperative thyroid function testing to be performed, because they contain thyroid tissue and postoperative hypothyroidism.

Cervical teratomas can typically and promptly be removed with surgery. Surgery is efficient and has a positive long-term effect. In addition, close monitoring is required in preventing the emergence of any post-operative respiratory distress. [10], [11], [12], [13], [14].

## Conclusion

Infantile cervical teratomas are rare and mostly benign tumors. On the other hand, certain thyroid teratomas are considered as a malignant and risk for recurrence. The authors highlighted that pediatric cervical teratomas should be acknowledged in determining a differential diagnosis for cystic lesions in the head and neck region. For the excellent outcomes, complete resection is highly recommended. A constant and proper long-term follow-up is essential to monitor potential complications from the malignancy or the possibility of recurrence disease in the future.

## Patient consent

Written informed consent was obtained from the patient for the publication of this case report.
